# Good Slang or Bad Slang? Embedding Internet Slang in Persuasive Advertising

**DOI:** 10.3389/fpsyg.2019.01251

**Published:** 2019-06-07

**Authors:** Shixiong Liu, Dan-Yang Gui, Yafei Zuo, Yu Dai

**Affiliations:** Department of Marketing, College of Management, Shenzhen University, Shenzhen, China

**Keywords:** internet slang, eye tracking, luxury goods, product evaluation, advertisement

## Abstract

Internet slang is a new language with innovative and novel characteristics, and its use can be considered a form of creative advertising. Embedding internet slang into advertisements can thus enhance their creative quality and increase the attention paid to them. In this study, we examined the effect of the characteristics of internet slang on attention to advertisements, brand awareness, product evaluation, and attitudes toward advertising by conducting two empirical studies, one utilizing eye-tracking experiments and the other utilizing questionnaires. We found that using internet slang in advertising significantly increased audience attention compared with standard language but did not necessarily improve product evaluation and brand awareness for various types of goods. We discovered code-switching effects of psycholinguistics existed in standard language and its variant (internet slang). Our findings can guide advertisers in selecting the embedded language that can be effective in achieving their desired advertising effect. Our findings also indicate that the excessive use of internet slang may have a negative effect on brand and product evaluation.

## Introduction

As society and the economy continue to develop, internet slang has shifted from being a mode of communication to being an everyday language. People’s communicative behavior, language, and psychology have all been affected by the subtle influence of internet slang (Crystal, 2006). Corporations have also started employing internet slang in public communications. McDonald’s, for example, used the internet slang “么么哒” (*Mo Mo Da*, a mimetic word for kissing) to promote its “Ice Cream Day,” because this word expresses ideas such as cuteness, proximity, and delightfulness. Some internet slang originates from the news, movies, TV programs, or online videos. For example, a popular online video featuring a character from an American TV show saying the phrase “Cash me ousside, howbow dah” (“Catch me outside, how about that?”) went viral because of the strong accent and rebellious attitude of the character. On the internet, a catch phrase or an incident can be publicized overnight, such as the expression “prehistoric powers” introduced by the young Chinese swimming athlete Yuanhui Fu or the emerging blend “Brexit” referring to the UK public vote for departure from the European Union. Internet slang has attracted the attention of corporations and its widespread use continues to grow.

In this study, we posed the following question: Is internet slang suitable for every product? It is possible that overusing internet slang in advertisements may yield unfavorable results, although such slang might attract more attention compared with standard language (SL). Therefore, this study explored people’s attention and evaluations (such as product evaluation and brand awareness) when encountering internet slang in various types of advertisements for products. The study also addressed whether internet slang is always has a positive effect on such evaluations.

This research makes several notable contributions. First, it adds to the literature related to advertising effect of languages, our work demonstrated the complex effects of internet slang on advertisements. Second, this work examined the advertising effect of internet slang from the attention perspective according to code-switching theory by using embedded language and eye tracking. These findings enrich both language and advertising communication theories.

## Theoretical Framework

### Internet Slang

The emergence of internet slang is a result of language variation. Language variation is a core concept in sociolinguistics (Chambers, 2008) and a characteristic of language, which means there is more than one way of saying the same thing. Speakers may use distinct pronunciation (accent), word choice (lexicon), or morphology and syntax. In this research, internet slang is regarded as a variant of SL because it is normally related to word choice or morphology and syntax. Internet slang as a variant of SL (e.g., English, Chinese, and German) (Collot and Belmore, 1996) is informal, irregular, and dynamic.

Internet slang often borrows foreign words, dialects, digital elements, and icons; it also frequently integrates the use of paraphrasing, homonyms, thumbnails, reduplication, and other word formation methods and unconventional syntax (Kundi et al., 2014). Internet slang has gained a “novelty” effect through its anticonventional nature, which is why non-normativity is its defining characteristic. Compared with SL, internet slang has innovative and novel characteristics (Collot and Belmore, 1996), and its use in advertising is highly creative. Attention to advertisements has increased following improvements in creative quality (Pieters et al., 2002). For example, in tobacco advertisements, creative warnings attract more audience attention than regular warnings do (Krugman et al., 1994). Exciting visuals can increase the perception of creativity, which attracts more attention to advertisements (Hagtvedt, 2011).

Internet slang is novel, humorous, and interesting, and it possesses qualities that attract attention, particularly that of humor (Eisend, 2011). By contrast, SL is more credible than non-SL. For example, the use of a standard accent in advertisements can largely offset any geographic, racial, or product differences (Alcántara-Pilar et al., 2013); thus, a considerable number of studies have recommended the use of SL to improve the influence of communication. In our previous study, we observed that compared with advertisements that used SL, those that used internet slang attracted more attention (Liu et al., 2017). Furthermore, electroencephalography studies have demonstrated that the cognitive processing of internet slang yields a significant N400 component and may involve creative thinking (Zhao et al., 2017).

### Internet Slang, Embedded Language, and Code-Switching Theory

Internet slang is often used in combination with SL. For example, communication in SL is occasionally interspersed with some internet slang terms to increase the attractiveness. Accordingly, internet slang is used in everyday life in the form of an embedded language. The advertising tactic of “inserting a foreign word or expression into a sentence (e.g., into an ad slogan), resulting in a mixed-language message” is called code-switching (Luna and Peracchio, 2005a; Lin et al., 2017). We applied code-switching theory in this research because the use of internet slang in advertising results in a similar situation as that of using code-switching. First, internet slang differs from SL because of its salient features, such as creative use of punctuation (e.g., emoticons), use of initialisms, omission of non-essential letters, and substitution of homophones (Jones and Schieffelin, 2009). This distinctive style enables audiences to distinguish internet slang from SL when embedded in advertisements. For example, a study (Zhao et al., 2017) reported that the processing of internet slang involves a novel N400 and late positive component, which reflects the recognition of the novel meanings of internet slang through event-related potentials (ERPs). Second, the language schema of internet slang is different from that of SL. Internet slang is heavily used by young people in computer-mediated communications and is usually perceived as creative, interesting, and pop culture-related (Tagliamonte, 2016). However, for adults who mainly speak SL, internet slang is viewed as informal and extremely difficult to understand (Jones and Schieffelin, 2009). Thus, examining the use of internet slang in advertising from the perspective of code-switching is reasonable.

The Markedness Model (Myers-Scotton, 1993) has been used to explain the code-switching direction effect (Luna and Peracchio, 2005b). The linguistic term “markedness” is analogous to perceptual salience (Luna and Peracchio, 2005b). When an object or part of a message stands out from its immediate context, it becomes salient from the audience’s prior experience or expectation, or from foci of attention (Fiske and Taylor, 1984). In regard to code-switching, the Markedness Model suggests that individuals will switch languages or insert other-language elements into their speech so as to communicate certain meanings or group memberships. Another language element becomes marked because of its contrast with the listener’s expectation. Luna and Peracchio (2005b) further explained that a marked element is recognized by the parties involved in the exchange as communicating a specific intended meaning. Scholars have argued that in a code-switching situation, the language schema of the words embedded in a message is activated because such words are more salient or marked compared with the matrix language. Language schemata include individuals’ perceptions of the social meanings of the language, the culture associated with the language, attitudes toward the language, the type of people who speak the language, the contexts in which the language can be used, the topics for which the language is appropriate, and beliefs about how others perceive the language (Luna and Peracchio, 2005a,b). For example, Luna and Peracchio (2005a) found that the language schema of Spanish, a minority language in the United States, can be activated when Spanish words are embedded in an ad slogan written in English (and vice versa). We propose that internet slang and SL may have similar code-switching effects when they are mix-used in advertisements. Therefore, this research involved conducting two studies to investigate whether code-switching effects occur between internet slang and SL, although internet slang is a variant of SL instead of a foreign language.

We believe that when internet slang is embedded in SL, the novelty of advertisements can provide a refreshing change for the audience and thus more likely garner their attention. Using eye movement tracking, we aimed to study the advertising effects produced by the use of internet slang as an embedded language, determine whether the use of internet slang as an embedded language can attract more attention, and explore whether this can generate positive advertising effects in terms of product evaluation and brand awareness. We expected that internet slang leads to an increase in consumers’ attention toward products, but excessive internet slang in advertisement does not necessarily generate a positive effect:

**H1**: Embedded internet slang (EIL) (vs. SL) in advertisements results in an increased number of fixations and fixation time.

### Luxury and Necessity Goods

Consumers may prefer different advertisements for various types of products, such as those that are functional or hedonic (Drolet et al., 2007). Luxury brands are typically associated with social status, prestige (Han et al., 2010), and superior product quality (Zhan and He, 2012). Consequently, the purchase of luxury goods requires advertisements that resonate with the identity of consumers and thus attract their attention. Accordingly, SL can be reminiscent of a high value and trust level (Lin and Wang, 2016), however, internet slang is timeliness, brisk and civilian that more consistent with style of necessity goods, could make necessity goods vivid and brisk; these features may increase consumers’ evaluations for brand and product. On the other hand, advertisement using internet slang for luxury brands may not be very appropriate, internet slang’s brisk and civilian style do not match with nobility and credibility of luxury goods, thus may not be better than SL which is meet the expectation of high value and credibility (Lin and Wang, 2016). Moreover, overusing internet slang may result in frivolous feeling that would compromise the high quality which luxury goods state. Therefore, whether the use of EIL in advertisements for luxury and necessity goods generates different advertising effects is a subject that merits investigation. Moreover, there is a relative lack of empirical research on advertisements and on the effects of EIL and SL in advertisements for necessity and luxury goods.

**Figure 1:**
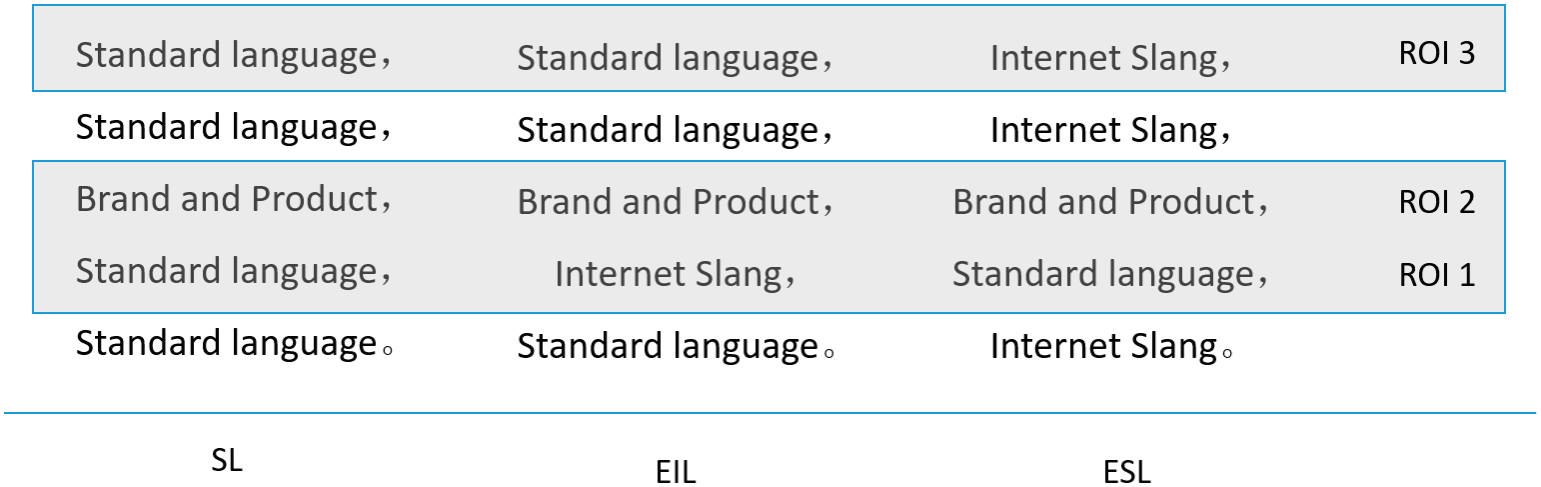
ROI zoning of SL, SIL, and ESL in study 1.

In this study, eye tracking was the primary means of measurement employed. We used eye tracking because its superior signal-to-noise ratio (relative to brain imaging) renders it more suitable for the study of attention when individuals evaluate various types of products and make a choice. We conducted two studies to empirically examine the effects of using internet slang as an embedded language in advertising copies, the audience’s attention when reading the copies, and the effect of different embedded language advertising formats on the audience’s product evaluation, brand awareness, and attitudes toward advertising. We also intended to test whether overusing internet slang in advertisements compromises the persuasive effect of the advertisements. Therefore, we designed another type of advertisement that comprised several internet slang words with embedded standard language (ESL). The difference between EIL and ESL is that the main body of ESL advertisement was used internet slang, one sentence using SL was embedded (see Figure 1); in the contrast, the main body of EIL advertisement was used SL, one sentence using internet slang was embedded (see Figure 1). ESL was designed to overuse internet slang. We hypothesized the following: (1) regarding advertising copies, advertisements of EIL would be more effective in attracting consumers’ attention compared with advertisements of SL or ESL and (2) the use of internet slang would attract different levels of attention and have distinct advertising effects depending on the type of product (necessity goods and luxury goods) for which it is employed. These hypotheses are outlined as follows:

**H2**: EIL in advertisements of luxury goods (vs. necessity goods) attracts more attention (an increased number of fixations and fixation time).

**H3a**: EIL (vs. SL) in advertisements of necessity goods results in increased product evaluation, brand awareness, and attitude toward advertisements.

**H3b**: EIL (vs. SL) in advertisements of luxury goods makes no significant difference in brand awareness, product evaluation, and attitude toward advertisements.

**H4**: ESL (vs. SL) in advertisements of luxury goods results in decreased product evaluation, brand awareness, and attitude toward advertisements.

## Pilot Study

Before conducting formal experiments, we first performed a pilot study on advertising language screening and advertising copy evaluation. The pilot study served two purposes. The first purpose was to confirm that the three language versions (SL, ESL, and EIL) used in subsequent studies would not exhibit semantic differences; accordingly, we could exclude alternative explanations of semantics. The second purpose was to ensure that the products and advertisements selected would not exhibit any distinct appeal.

To compile a list of internet slang words, we applied our screening process to select the 20 most searched terms in China on Baidu. The primary criteria established for this process were as follows: the term must be well known; its usage must be widespread; and it must not have negative connotations, rendering it suitable for the design of an advertising copy. The designed advertising copy covered necessity goods, such as mineral water, toothpaste, cooking oil, towels, and shampoo, and luxury goods, such as watches, cars, perfume, jewelry, and leather items. Finally, the materials of the pilot study included 30 advertisements of five necessity goods (each product included three different language versions of SL, ESL, and EIL) and five luxury goods (each product included three different language versions of SL, ESL, and EIL). To exclude the influence of prior knowledge, all brands of products used in the advertisements were fabricated and not similar to any real brand, for excluding the influence of prior knowledge.

We first divided the advertising language into 10 groups for various products; each group contained three types of advertising language. Subsequently, the participants were asked to view advertisements in the three types of language (SL, ESL, and EIL) and to evaluate whether there were differences in semantics among the three language versions (SL, ESL, and EIL), which were measured on a five-point Likert scale. The findings revealed that for each product, the three versions of advertising language (SL, ESL, and EIL) yielded no semantic differences. Specifically, for necessity goods, such as toothpaste [mean = 2.43, standard deviation (SD) = 1.43], mineral water (mean = 2.33, SD = 1.30), cooking oil (mean = 2.60, SD = 1.43), towels (mean = 2.43, SD = 1.46), and shampoo (mean = 2.27, SD = 1.36), the means were all below the median (3). For luxury goods, such as watches (mean = 2.13, SD = 1.07), cars (mean = 2.40, SD = 1.33), perfume (mean = 2.63, SD = 1.52), jewelry (mean = 2.50, SD = 1.33), and leather items (mean = 2.33, SD = 1.16), the means were all below the median (3). The participants were 30 undergraduate students from the Shenzhen University. The results indicated that there were no significant semantic differences between the three versions of advertising language, signifying that our study would not be affected by semantic differences.

We recruited an additional group of 30 participants for the pilot study, all of whom were young people including university students and new employees. Concurrently, to avoid other differences caused by the copy used, manipulative variables were used to rate the responses regarding the rational and emotional appeal of the same 30 advertisements. Resnik and Stern proposed a standard definition of rational appeal based on 11 classification criteria: price, quality, characteristics, ingredients, purchase time and location, means of promotion, trial, function, packaging, guarantees, and novelty (Resnik and Stern, 1977). Sciulli and Lisa (1998) proposed an emotional appeal scale comprising the following items: happiness, fear, joy, anger, interest, disgust, sadness, surprise, and numerous other emotional experiences. Therefore, these classification criteria were adopted in the pilot study. To measure advertising appeal, we selected four items (quality, ingredients, guarantees, and novelty) from the rational appeal scale and four items (happiness, interest, disgust, and sadness) from the emotional appeal scale. The participants were asked to evaluate these items for the 30 advertisements.

For all statistical analyses performed using SPSS version 24.0 (SPSS, Inc., Chicago, IL), the significance level was set to 0.05. Analyses of variance (ANOVAs) were conducted using language type (SL, ESL, and EIL) by product type (necessity goods vs. luxury goods) as within-subjects factors. For the formal experiment, we selected the copy according to the ratings received in the pilot experiment. We conducted an ANOVA using language type (SL, ESL, and EIL) and product type (necessity and luxury) as the independent variables and rational and emotional appeal scores as the dependent variables. No significant main effects of language version were observed for rational appeal [*F*(1, 29) = 1.616, *p* = 0.199] or emotional appeal [*F*(1, 29) = 2.247, *p* = 0.106]. Furthermore, the main effects of product categories revealed no significant differences for rational appeal [*F*(1, 29) = 1.277, *p* = 0.259] or emotional appeal [*F*(1, 29) = 0.092, *p* = 0.762]. Moreover, the two-way interaction was not significant for rational appeal [*F*(1, 29) = 0.066, *p* = 0.939] or emotional appeal [*F*(1, 29) = 0.266, *p* = 0.767]. Therefore, the advertisements in three languages for both necessity goods and luxury goods did not differ in terms of rational and emotional appeal scores. The experimental materials were thus suitable for formal experimental study to explore the effect of advertising language versions on product evaluation, brand awareness, and attitude toward advertisements.

## Study 1

### Participants

In total, 120 healthy volunteers (71 female individuals; mean age: 22.42 years) from the Shenzhen University, China, participated in the experiment, although six were subsequently excluded because of recording errors and severe artifacts in the data; the included participants took MBA classes and had an independent income and several years of work experience. A 2 (product type: necessity goods vs. luxury goods) × 3 (language type: SL, ESL, and EIL) between-subjects design was employed (factors were not significantly correlated). All participants were right-handed, had normal vision (with or without correction), reported no history of affective disorders or neurological diseases, and did not regularly use medication. All participants provided written informed consent before the experiment, and the study protocol was approved by the Local Ethics Committee of Shenzhen University. All methods were conducted in accordance with the approved protocol.

### Procedures

In this experiment, we used the ASL-D6 eye-tracking system developed by the Applied Sciences Laboratory in the United States. This system has outstanding capturing and contract capabilities, can rapidly and accurately compensate for head movement, and can provide instant feedback during the tracking process. Thus, this system met the requirements of this experiment. After the initiation of the experiment, the screen displayed an advertising copy that was viewed by the participants. Upon completion of the experiment, the participants’ eye movement data and basic information were stored; the participants were then asked to complete a questionnaire regarding the content they had viewed.

Three types of languages were used for the advertisements, namely SL, SL embedded with internet slang, and internet slang embedded with SL. Thirty advertisements of five necessity goods (each product included the three different language versions of SL, ESL, and EIL) and five luxury goods (each product included the three different language versions of SL, ESL, and EIL) were used. As illustrated in Figure 1, each advertisement contained five short sentences. Each advertisement was presented for 12 s to the participants. The study sequence was counterbalanced. The condition “SL” means that all sentences in the advertisement used SL. The condition “EIL” means that the main body of the advertisement was SL, but one sentence using internet slang was embedded; the non-embedded language was one sentence that did not contain internet slang, brand, or product name. The condition “ESL” means that the main body of the advertisement was internet slang, but one sentence using SL was embedded; the non-embedded language was one sentence that did not contain SL, brand, or product name. Figures on the advertisement copy with embedded language were then compared and adjusted to ensure that SL was embedded with internet slang (and vice versa), that the corresponding regions of interest (ROIs) of each figure were the same, and that brand names were placed in the same location.

After the eye movement experiment, the participants answered a questionnaire on the advertising copy. Experimental stimuli were divided into three ROIs (Figure 1). An ROI is a specific region presented to participants for visual stimulation. To perform an intergroup comparison, the selected ROI for the same types of products was same placement of the embedded and non-embedded languages within the ROI. ROI1 contained the embedded language; ROI2 contained the brand and product name; and ROI3 contained the non-embedded language.

We selected three commonly used measures to evaluate attention to advertisements: fixation time (Wedel and Pieters, 2000; Rayner et al., 2001; Decrop, 2007), number of fixations (García et al., 2000; Wedel and Pieters, 2000; Wang and Day, 2007), and pupil diameter (Krugman et al., 1994). Fixation time is the length of time a participant spends viewing the target zone, and it represents the amount of information they have processed in the zone. The longer the time is, the deeper the information processing in a specific area is. The number of fixations is a measure of the frequency of fixation in a zone by a participant; it represents the amount of information the participant has processed in the zone. The higher the number of fixations is, the greater the attention paid to the information in a specific zone is. The pupil diameter measures the size of the pupil; it represents the level of interest a participant shows in a specific zone. When the pupil diameter is enlarged, it implies the participant is viewing a zone that interests him or her.

Because arousal plays a vital role in cognitive tasks (Schimmack and Derryberry, 2005; Dresler et al., 2009) by stimulating audience attention, the level of arousal was used as a control variable. According to Massar et al. (2011), the question whose answer ultimately determines the level of arousal is “Were you calm when you viewed this ad?” The other control variables measured were follows: familiarity with internet slang, attitude toward the internet slang used, and product preferences.

### Results

For all statistical analyses performed using SPSS version 24.0 (SPSS, Inc., Chicago, IL), the significance level was set to 0.05. Post-hoc tests for multiple comparisons were corrected using the Bonferroni method. Significant interactions were analyzed through simple-effect models. ANOVAs were conducted using language type (SL, ESL, and EIL) by product type (necessity goods vs. luxury goods) as between-subjects factors.

**Figure 2:**
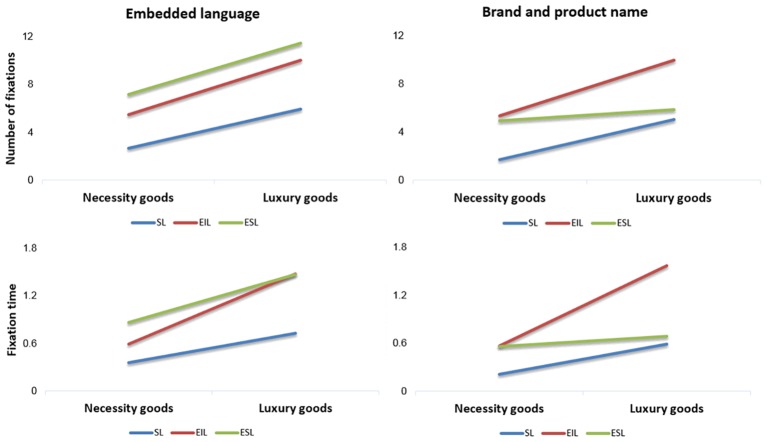
Number of fixations and fixation time for standard language (SL), embedded standard language (ESL), and embedded internet slang (EIL) for necessity goods and luxury goods in study 1.

Statistical results (Figure 2) revealed that (1) in ROI1 (embedded language), language type [*F*(2, 110) = 5.871, *p* = 0.004] and product type [*F*(1, 110) = 12.185, *p* = 0.001] had significant main effects on fixation time; however, the interaction between language type and product type exhibited no significant effect [*F*(2, 110) = 1.153, *p* = 0.319]. Regarding the product type, the fixation time on necessity goods was shorter than that on luxury goods. Concerning language type, the fixation time on ESL was the longest, followed by that on EIL and then that on SL. All means and SDs are presented in Table 1.

**Table 1:** Mean and SD for standard language (SL), embedded standard language (ESL), and embedded internet slang (EIL) for necessity goods and luxury goods in study 1.

ROI	Language		Number of fixations		Fixation time	
			Mean	SD	Mean	SD
Embedded language	SL	Necessity goods	2.65	2.09	0.36	0.46
		Luxury goods	5.96	4.99	0.73	0.69
	ESL	Necessity goods	7.19	5.24	0.87	0.85
		Luxury goods	11.46	7.65	1.47	1.48
	EIL	Necessity goods	5.49	3.21	0.59	0.54
		Luxury goods	10.04	6.84	1.48	0.84
Brand and product name	SL	Necessity goods	1.73	1.87	0.22	0.46
		Luxury goods	5.04	4.34	0.59	0.71
	ESL	Necessity goods	4.94	3.39	0.56	0.62
		Luxury goods	5.86	3.88	0.69	0.77
	EIL	Necessity goods	5.37	3.81	0.57	0.60
		Luxury goods	9.95	5.96	1.57	1.15

Language type [*F*(2, 111) = 9.944, *p* < 0.001] and product type [*F*(1, 111) = 14.148, *p* < 0.001] exerted significant main effects on number of fixations; however, the interaction between language type and product type displayed no significant effect [*F*(2, 111) = 0.226, *p* = 0.798]. Regarding the product type, the number of fixations on necessity goods was lower than that on luxury goods. Concerning the language type, the number of fixations on ESL was the highest, followed by that on EIL and then that on SL. All means and SDs are presented in Table 1.

(2) In ROI2 (brand and product name), language type [*F*(2, 110) = 7.998, *p* = 0.001] and product type [*F*(1, 110) = 12.335, *p* = 0.001] had significant main effects on fixation time, and the interaction between language type and product type exerted a significant effect on fixation time [*F*(2, 110) = 4.298, *p* = 0.016]. The fixation time on necessity goods was shorter than that on luxury goods. Regarding the language type, the fixation time on EIL was the longest, followed by that on ESL and then that on SL. These results suggest that EIL attracts more attention to brand and product names. Furthermore, the results of simple-effect tests showed that for necessity goods, the use of EIL and ESL had no effect (but they performed better than SL alone), whereas for luxury goods, the use of EIL and ESL had a significant effect. Therefore, EIL outperformed SL, whereas ESL and SL did not differ in performance. All means and SDs are shown in Table 1.

Language type [*F*(2, 111) = 11.615, *p* < 0.001] and product type [*F*(1, 111) = 16.197, *p* < 0.001] had significant main effects on the number of fixations; however, the interaction between language type and product type exhibited no significant effect [*F*(2, 111) = 2.490, *p* = 0.088]. Concerning the product type, the number of fixations on necessity goods was lower than that on luxury goods. In terms of the language type, the number of fixations on EIL was the highest, followed by the number of fixations on ESL and SL. All means and SDs are listed in Table 1.

### Summary

Our results reveal that the type of language used in the advertisements significantly influenced the participants’ attention to both necessity and luxury goods. Internet slang in the advertisements was proved to be eye-catching, and ESL attracted much more attention than SL and EIL did in the ROI of embedded language. However, in the ROI of brand and product name, EIL attracted more attention than SL and ESL did.

## Study 2

### Participants

A total of 900 healthy volunteers (420 female individuals, mean age: 23.68 years; 580 student samples and 271 non-student samples) from the Shenzhen University, China, participated in the experiment, of whom 49 were excluded because of incorrectly answered questionnaires; therefore, the final sample comprised 580 students and 271 nonstudents. A 2 (product type: necessity goods vs. luxury goods) × 3 (language type: SL, ESL, and EIL) between-subjects design was employed (factors were not significantly correlated). All participants were right-handed, had normal vision (with or without correction), reported no history of affective disorders or neurological diseases, and did not regularly use medication. All participants provided written informed consent before the experiment, and the study protocol was approved by the Local Ethics Committee of the Shenzhen University. All methods were conducted in accordance with the approved protocol.

### Procedures

The products and advertising copy employed in this experiment were the same as those used in study 1. We created an online survey on WJX,^1^ a widely used online survey platform in China, to measure all the variables for experiments. Online surveys are usually subject to concerns such as an insufficient amount of time spent on questions and multiple questionnaires being completed by the same individual. The WJX survey platform avoids these problems by setting a minimum duration required to complete a questionnaire and by preventing users with the same IP address or device from participating multiple times.

We combined the scales developed by Gardner et al. (1985) and Huang et al. (2006) to determine five questions used to measure attitudes toward advertisements. Brand awareness is based on the brand equity model (Keller, 1993) and includes both brand recognition and brand recall. Brand recognition refers to aided brand awareness, whereas brand recall refers to unaided brand awareness. To measure product evaluation, Dodds et al. (1991) proposed the use of perceived product quality, perceived product value, and purchase intent. We tested all three measures (*p* < 0.001) using a univariate analysis, and their component reliability was higher than the recommended standard of 0.6. Finally, the following measures of product evaluation were used: perceived product quality (quality, reliability, and durability), perceived product value (cost effectiveness, acceptability, and value for money), and purchase intent (purchase intent and considering purchase). The control variables in our study were as follows: (1) familiarity with internet slang, (2) attitude toward the internet slang, (3) product preferences, and (4) arousal. We selected all these control variables as covariates in the ANOVA.

### Results

For all statistical analyses performed using SPSS version 24.0 (SPSS, Inc., Chicago, IL), the significance level was set to 0.05. Post-hoc tests for multiple comparisons were corrected using the Bonferroni method. Significant interactions were analyzed through simple-effect models. ANOVAs were conducted using language type (SL, ESL, and EIL) by the product type (necessity goods vs. luxury goods) as between-subjects factors.

**Figure 3:**
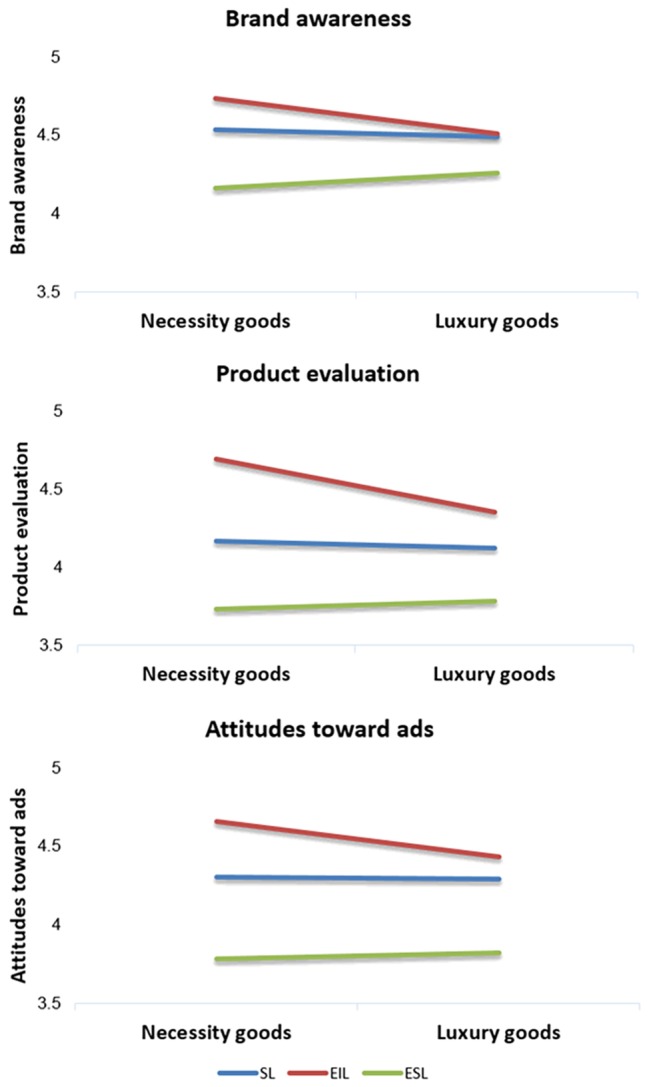
Brand awareness, product evaluation, and attitude toward advertisements for necessity goods and luxury goods in study 2.

Statistical results (Figure 3) indicated that the language type had a significant main effect [*F*(2, 844) = 8.767, *p* < 0.001] on brand awareness, although the main effect of product type was not significant [*F*(1, 844) = 0.623, *p* = 0.430]. The interaction between the language type and product type exhibited no significant effect [*F*(2, 844) = 1.888, *p* = 0.152]. Overall, the brand awareness of EIL was the highest and was significantly higher than that of ESL. All means and SDs are presented in Table 2.

**Table 2:** Mean and SD for standard language (SL), embedded standard language (ESL), and embedded internet slang (EIL) for necessity goods and luxury goods in study 2.

Language		Attitudes toward ads		Brand awareness		Product evaluation	
		Mean	SD	Mean	SD	Mean	SD
SL	Necessity goods	4.30	0.82	4.53	0.79	4.17	0.79
	Luxury goods	4.29	0.88	4.49	0.97	4.13	0.88
ESL	Necessity goods	3.78	1.31	4.17	1.26	3.74	1.21
	Luxury goods	3.82	1.39	4.26	1.33	3.79	1.38
EIL	Necessity goods	4.66	0.99	4.74	0.91	4.70	0.90
	Luxury goods	4.44	1.04	4.51	0.98	4.36	1.04

Language type [*F*(2, 845) = 47.125, *p* < 0.001] and product type [*F*(1, 845) = 6.163, *p* = 0.013] had significant main effects on product evaluation; however, the interaction between language type and product type exhibited no significant effect [*F*(2, 845)= 1.888, *p* = 0.529]. Overall, the product evaluation of EIL was the highest and was significantly higher than that of ESL. All means and SDs are shown in Table 2.

Language type had a significant main effect [*F*(2, 845) = 34.368, *p* < 0.001] on attitudes toward advertisements; however, product type exhibited no significant main effect [*F*(1, 845) = 0.747, *p* = 0.388]. The interaction between language type and product type exhibited no significant effect [*F*(2, 845) = 1.183, *p* = 0.307]. Overall, the brand awareness of EIL was the highest and was significantly higher than that of ESL. All means and SDs are presented in Table 2.

**Figure 4:**
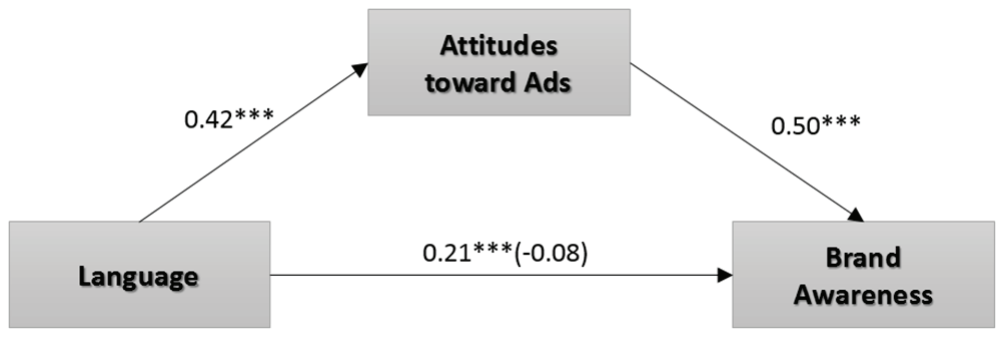
Mediation of language type to brand awareness through attitudes toward advertisements. *p < 0.05; **p < 0.01; ***p < 0.001.

The observed mediational relationship was confirmed by a bootstrapping analysis (bias-corrected; 10,000 samples), in which the 95% confidence interval in the indirect effect did not include zero (0.05, 0.90). Bootstrap results revealed that the indirect effect was significant (*p* < 0.001) and that attitudes toward advertisements mediated the effect of language type on brand awareness (Figure 4). These results suggest that the advertisements that used EIL performed better than those that used ESL and SL regarding brand awareness, product evaluation, and attitudes toward advertisements.

### Summary

Our results reveal that EIL advertisements had higher ratings on brand awareness, product evaluation, and attitudes toward advertisements than SL and ESL advertisements did. Compared with EIL advertisements, ESL advertisements had the lowest of all ratings, even lower than those of SL advertisements. This indicates that the excessive use of internet slang may have a negative effect on brand and product evaluation. For luxury goods, internet slang did not generate a positive effect on brand awareness compared with SL.

## Discussion

Changes in languages used in advertising can affect the market value of corporations. Advertising languages that are outstanding or have gained consumers’ recognition exert significant and positive effects on the development and market value of corporations (Mathur and Mathur, 1995). Advertisements that focus on consumer recognition and use modern internet slang may exert a positive effect on both a firm and its product(s). We argue that the effect of internet slang on advertisement is complex; it depends on the types of products and the embedding style. Our findings indicate that advertisements with internet slang are not always attractive, and the excessive use of internet slang may have a negative effect on brand and product evaluation.

### Theoretical Contribution and Implications

#### Code-Switching Theory

According to Ahn et al. (2017), code-switching is a mixed-language approach and is often used to target consumers with knowledge of two languages. Code-switching refers to the insertion of linguistic elements of one language into another language (Grosjean, 1982). An example of code-switching is inserting an English word into a Korean sentence (Ahn et al., 2017). However, most studies examining the effect of code-switching on processing ads have been undertaken in the United States by focusing on the mixed use of Spanish and English languages (Luna and Peracchio, 2005a,b; Bishop and Peterson, 2010). Ahn et al. (2017) suggested that additional research is warranted in other regions where code-switching occurs between languages other than English and Spanish.

Our study was undertaken in the China market. Chinese language is a character-based writing system as well as a meaning-based writing system, whereas English is a sound-based writing system and an alphabetic writing system (Cook and Bassetti, 2005). Our results indicate that code-switching effects occur not only in a sound-based and alphabetic writing system but also in a character-based and meaning-based writing system. Therefore, these findings extend the external validation of code-switching theory.

Furthermore, in this study, we investigated SL (Mandarin) and its variant (internet slang), and the results demonstrate that code-switching theory is also effective to SL and its variant. Specifically, the validation of code-switching is further extended because previous research has mainly focused on the mixed use of two different languages (Bishop and Peterson, 2010; Ahn et al., 2017). Finally, by empirically investigating the role of code-switching in advertising effectiveness, the findings of this study provide theoretical and practical implications regarding the code-switching approach for researchers and advertisers.

#### Novelty and Attention of Internet Slang

SL and internet slang have distinct characteristics. When advertisements use SL, a feeling of standardization and strictness is induced (Vignovic and Thompson, 2010); by contrast, when advertisements use internet slang, consumers identify the signals sent by the language, such as novelty or trendiness (Collot and Belmore, 1996; Crystal, 2006), with their own personalities, making them feel closer to the brand and generating a more favorable emotional experience. Therefore, compared with advertisements in SL, advertisements embedded with internet slang highlight the fun and fresh characteristics of such slang; consequently, people form more positive attitudes toward such advertisements.

Liu et al. (2013) reported that advertisements in Cantonese and Mandarin have different advertising effects, and Henderson et al. (2004) revealed that trademarks in standard and handwritten typefaces can leave different impressions. Thus, different effects are exerted depending on how advertising language is presented. Exciting advertisements evoke positive emotions from consumers, and the consumers associate these with the product (Eunsun et al., 2005). Internet slang is generally considered to be humorous, fun, and exciting (Collot and Belmore, 1996). The employment of internet slang in advertising copies exerts a “novelty” effect on the corresponding advertisement; therefore, attention increases as advertisements become more creative (Pieters et al., 2002). A novel advertising language that is creative can attract more attention, which is in line with the findings of our previous study. The novelty, humor, and fun characteristics of internet slang are evident when EIL appears in advertisements (Pieters et al., 2002); thus, advertisements in SL that are embedded with internet slang can attract more attention compared with other advertisements.

Peng et al. (2017) reported that consumers believed the exposure time to internet slang was longer than that to SL, although internet slang and SL as stimuli lasted for the same period in their experiment. A possible explanation for these results is that consumers have to spend more resources on processing internet slang. The results of our eye-tracking experiments support this supposition, and we discovered that EIL (vs. SL) in advertisements results in an increased number of fixations and a longer fixation time.

The eye-catching ability of internet slang is attributable to its higher amount of information and greater association for consumers, which thus signifies that internet slang requires more time to process. A recent ERP study on internet slang indicated that the information processing fluency of internet slang is much lower than that of SL (Zhao et al., 2017). This finding is also supported by our eye-tracking experiments; more attention is paid to internet slang. The reason for this outcome requires elucidation. This outcome can be explained by the novelty of internet slang, which originates from pop culture. Previous research suggested that internet slang is considered novel and innovative (Hagtvedt and Patrick, 2008). This is because internet slang is generally created in a creative and innovative manner. Thus, since its creation, internet slang has been accepted and spread rapidly and extensively (Liu et al., 2017). Furthermore, Zhao et al. (2017) argued that internet slang is perceived through creative information processing; this perception process reflects the recognition of the novel meanings of internet words as well as the integration of novel semantic processing.

The innovativeness of language has a crucial advertising effect (Eisend, 2011). Internet slang is inherently creative, and the creativity of internet slang has a positive influence on consumers’ perception of advertisements (Karson and Fisher, 2005). Specifically, Eisend (2011) suggested that the innovativeness of internet slang can elicit consumers’ perception of an ad’s innovativeness. This thus explains our finding that internet slang used in advertisements had positive effects on product evaluation, brand awareness, and attitude toward advertisements.

#### Complex Effect of Internet Slang on Various Types of Products

Necessity goods are indispensable for the daily lives of consumers and are extremely practical (Chen and Wang, 2012); consumers purchase such goods to fulfill their daily needs. Necessity goods are relatively cheap, are only slightly affected by information, and do not require extra information processing on the part of the consumer. Consumers can easily develop brand loyalty toward necessity goods in a way that transforms into habitual purchasing behavior (Monle and Tuen-Ho, 2003). In the ROIs of brand names of necessity goods in this study, EIL and ESL did not elicit distinct levels of attention (but both of them outperformed SL), indicating that internet slang helps increase consumers’ attention to brands of necessity goods. For luxury goods, the effects of EIL and ESL differed significantly; EIL outperformed SL, but ESL and SL did not differ in performance. These findings indicate that the excessive use of internet slang (advertising copy in ESL) does not increase audience attention to brands of luxury goods.

Luxury goods are subject to a high perceived risk; thus, information must be processed more carefully. In contrast to the level of information processing necessary for necessity goods, information on luxury goods requires in-depth processing. When a product becomes a luxury good, the use of SL in advertisements prompts consumers to associate the advertised products with high quality because SL is associated with high value and credibility (Lin and Wang, 2016) and serves as the principal language with rigor and reliability (Yip and Matthews, 2006). Therefore, appropriately embedding internet slang can increase attention to a brand. However, the use of inappropriate internet slang would not achieve positive advertising effects.

Our study indicates that because of its high levels of creativity and timeliness, internet slang may temporarily increase audience attention to advertising language, but it cannot produce the same effect on higher status products (such as luxury goods). Furthermore, an excessive use of internet slang may cause the audience to feel frivolous, which damages the trust consumers have in a brand or product. For example, a highly trusted advertising language generates better results (Kronrod et al., 2012). The second experiment also showed that in terms of brand awareness and product evaluation, advertising copies in ESL had the lowest scores; the conventional use of SL for advertising copies can thus yield superior performance compared with the extensive use of internet slang for advertising copies.

### Practical Implications

The rapid spread of internet platforms means that internet slang can become a social buzzword under certain circumstances (Sun et al., 2011). Once internet slang gains public recognition and spreads at an extremely rapid rate, numerous corporations will begin to integrate it into their advertising copies. In practice, the use of internet slang requires careful consideration by marketing practitioners. Copies in internet slang can increase an audience’s attention, but they may also weaken their attention to other elements of the same advertisement. Although internet slang can significantly enhance product evaluation, it may undermine advertising reliability. Marketing practitioners should use internet slang based on their communication objectives to produce effective results. In addition, rather than simply following the current internet slang trends, marketing personnel should employ differentiated advertising strategies depending on the type of product to help align the implemented advertising copy with that product.

### Limitation and Future Direction

Our work has a few limitations, which opens up avenues for future research. First, creativity is one of the major features of internet slang, but it was not measured in our research. Novelty and creativity may be alternative explanations for the positive effect of EIL on attention and higher evaluations for advertisements and brand. Future studies should focus on the connection between attention and the creativity and novelty features of internet slang. Second, notably, novelty and creativity cannot explain the negative effect of ESL. The excessive use of internet slang possibly leads to frivolousness, vulgarity, and incredulity about an advertisement, particularly for luxury goods that exhibit strengthened superiority and dignity. Future studies could examine the various effects and corresponding mechanisms of EIL and ESL on advertisements.

Third, the process of code-switching costs more attention resources. Luna and Peracchio (2005a) argued that, when individuals direct their attention to the codeswitched word, they will activate the language schema to which that word belongs and become aware of the social meaning carried by that language. The language schema associated with the code-switched term is subject to a high degree of elaboration because of the markedness of the term (Johnston et al., 1990). However, more attention and high degree of elaboration do not necessarily mean EIL and ESL are confusing; in fact, code-switching is generally socially motivated and is rarely a sign of a lack of fluency in either language (Grosjean, 1982; Luna and Peracchio, 2005b). There might be some differences of how easy to understand the ads among SL, EIL, and ESL, future studies should add the items that measure how easy (or hard) to understand different advertisements.

Timeliness of internet slang is another interesting feature that we did not examine in the current research. Internet slang displays strong timeliness; the novelty and creativity of internet slang may decrease with time, and the corresponding positive effect may also decline. Finally, the current research examined the code-switching effect between SL and its variant for Chinese advertisements; however, whether it exists in other languages (such as English or Spanish) should be further examined by future studies.

## Conclusion

We examined the effect of internet slang on attention to advertisements, product evaluation, and advertising attitude by conducting two empirical studies, one of which involved eye-tracking experiments and the other applied questionnaires. The results show that advertisements in EIL generated positive brand awareness and product evaluation and also increased advertising attention. Our findings reveal the complex effects of internet slang on advertisements and extend the external validation of code-switching theory. These findings can guide advertisers in selecting an embedded language that can be effective in achieving their desired advertising effect. To attract an audience’s attention, the use of EIL can help consumers experience the creativity of internet slang, thereby forming positive brand awareness and product evaluation. However, for different types of products that address dissimilar needs, practitioners should avoid the extensive use of internet slang once they have decided which advertising strategy and advertising copy they will use, particularly in relation to luxury goods. They can decide to use SL or embed a small portion of internet slang into their advertisements.

## Ethics Statement

This study was carried out in accordance with the recommendations of Local Ethics Committee of Shenzhen University with written informed consent from all subjects. All subjects gave written informed consent in accordance with the Declaration of Helsinki. The protocol was approved by the Local Ethics Committee of Shenzhen University.

## Author Contributions

SL and D-YG conceived and designed the experiments. SL, YD, and YZ performed the experiments. D-YG and YZ analyzed the data. D-YG and SL wrote the manuscript. SL and D-YG contributed materials and analysis tools. SL provided lab equipment for running the study.

### Conflict of Interest Statement

The authors declare that the research was conducted in the absence of any commercial or financial relationships that could be construed as a potential conflict of interest.

## Footnotes

^1^https://www.wjx.com/
